# Could cytokine release syndrome induce acute myelofibrosis in CD19 chimeric antigen receptor T cells therapy?

**DOI:** 10.1080/21655979.2020.1791597

**Published:** 2020-08-10

**Authors:** Xun Lai, Yun Yan Sun, Lung Ji Chang, Yu Ru Ma, Xue Zhong Gu, Xiang Mei Yao, Bo Nie, Yan Wen, Xue Mei Zhang, Ya Xian Jiang, Hui Yang, Li Qun Yu, Ming Jing Fang, Ling Wang, Xue Yuan Bo

**Affiliations:** a Department of Hematology, The Third Affiliated Hospital of Kunming Medical University (Tumor Hospital of Yunnan Province), Kunming, Yunnan, China; b Shenzhen Geno-Immune Medical Institute, Shenzhen, China; c The First Affiliated Hospital of Kunming Medical University, Kunming, Yunnan, China; d Department of Hematology, The First People’s Hospital of Yunnan Province, Kunming, Yunnan, China; e Department of Hematology, The First Affiliated Hospital of Kunming Medical University, Kunming, Yunnan, China; f Department of Clinical Laboratory, The First People’s Hospital of Yunnan Province, Kunming, Yunnan, China; g Department of Pathology, The First People’s Hospital of Yunnan Province, Kunming, Yunnan, China; h The Medical University of Dali, Dali, Yunnan, China; i Cancer Biotherapy Center, The Third Affiliated Hospital of Kunming Medical University (Tumor Hospital of Yunnan Province), Kunming, Yunnan, China

**Keywords:** CAR-T, CRS, BMF, AMF, B-ALL

## Abstract

CAR-T cells therapy can give rise to most common and concerning two side effects – cytokine release syndrome (CRS) and neurotoxicity. But in our CD19 CAR-T cells therapy clinical trial, we observed 1 out of 17 patients with B-cell acute lymphoblastic leukemia (B-ALL) developed acute myelofibrosis(AMF) after grade IV CRS post to the CD19 CAR-T cells therapy. This finding suggests that the CAR-T cells therapy may have rare and serious AMF, which we should pay important attention to.

Trial registration:NCT02968472. Registered 18 November 2016 – Retrospectively registered, https://clinicaltrials.gov/ct2/show/NCT02968472

## Introduction

Chimeric antigen receptor (CAR) T cells therapy is an effective method for treating certain cancers. CARs are normally designed to recognize those antigens that are highly expressed on malignant cells [1,2]. The most compelling success of CAR-T cells therapy has been achieved in CD19-specific CAR-T cells for R/R B-cell acute lymphoblastic leukemia (B-ALL) with a complete remission (CR) rates of 70 ~ 94% [2,3]. However, life-threatening adverse events, including cytokine release syndrome (CRS) and neurotoxicity [4], can be major complications that lead to treatment failure. Current treatment strategy on CRS has been working on improving the safety of CAR-T therapy.

We developed one center, single-arm, phase II CD19 CAR-T cells clinical trial for relapsed/refractory B-cell ALL patient from March 2015 to December 2018. The CAR containing CD19-specific scFv fused withCD28-CD27-CD3ζ signaling domains and an inducible Caspase 9 motif (scFv/CD28/CD27/CD3ζ-iCasp9), was chemically synthesized and cloned into pTYF transduced vector with a human EF1α promoter. In clinical trials, we observed 1 out of 7 grade III–V CRS patients in 17patients with B-cell ALL developed Acute myelofibrosis (AMF) post CD19 CAR-T cells therapy. This is a rare situation and the mechanism remains elusive. Due to the severity of the adverse event, further investigation needs to be given for better care of CAR-T therapy.

## Case

**Figure 1.**
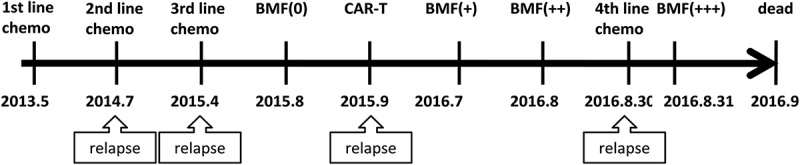
Therapy and bone marrow fibrosis (BMF) diagnosis line.

A 36-year-old man who was diagnosed with Philadelphia chromosome negative (Ph-) B-ALL. The JAK2 MPL and CALR mutation tests were negative. The case disease course as Figure 1. Before CAR-T therapy he received three lines of chemotherapy and achieved CR each time then relapsed eventually. Because the Patient had no suitable donor for stem cell transplantation then he didn’t receive allogeneic peripheral blood stem cell transplant. At the third relapsed, the B lymphoid blasts made up 25% in the marrow. He then received lympho-depletion chemotherapy with cyclophosphamide 40 mg/kg × 2d (day −3 to −2), followed by an infusion of autologous CD19 CAR-T cells(day 0). He developed grade IV CRS and presented as fever, hypotension on day 4. The observed highest plasma level for IL-6 and TNF-α were 399.5 pg/ml and 22.0 pg/ml, respectively. After about two months of CAR-T cells infusion, the marrow sample indicated there was no evidence of B-ALL minimal residual disease by Flow Cytometry (FCM), he achieved CR.

**Figure 2.**
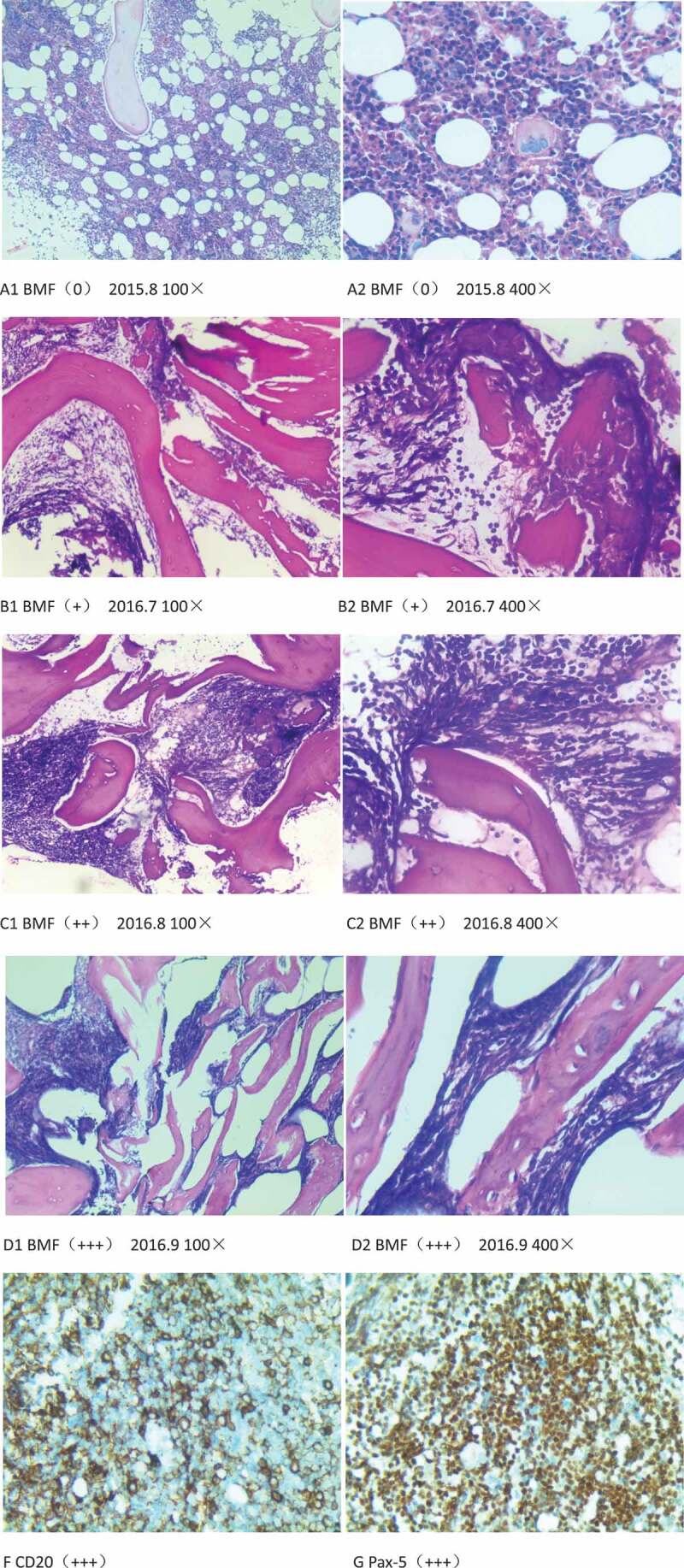
(a-d), (h) & (e) and reticulin stain of bone marrow fibrosis (BMF). (f,g), immunohistochemical (IHC) staining.

We performed the bone marrow biopsy before and after CD19 CAR-T cells infusion and monitored the status of the bone marrow. Interestingly, we observed the BMF progressively deteriorating from normal to grade III (Figure 1). Before CD19 CAR-T cells therapy or the third leukemia relapsed, the bone marrow biopsy showed normal (Figure 2(a)). After 10 months (2016.7) of CAR-T cells infusion, the bone marrow biopsy showed grade I BMF (Figure 2(b)). At 2016.8, the bone marrow biopsy showed BMF progressively deteriorating to grade II BMF (Figure 2(c)). At the end of August of 2016, again biopsy showed BMF progressively deteriorating to grade III BMF (Figure 2(d)). At this time, leukemia relapsed the fourth time, the result of blood showed pancytopenia (CBC: hemoglobin, 73 g/L; mean corpuscular volume, 98.2 fL; absolute neutrophil count, 0.57 × 10^9^/L; platelets, 52 × 10^9^/L). he received one cycle chemotherapy but it could not control the disease and he died.

At the last leukemia relapsed, peripheral blood mononuclear cells FCM was performed and it showed that the patient was still having B-ALL. The last bone marrow biopsy immuno histo chemical staining also showed that the patient was still having B-ALL, the result were CD19 (few cells +), CD10 (+++), CD7 (-), CD3 (-), TdT (++), CD20 (+++)(Figure IIF), Pax-5 (+++)(Figure 2(g)).

(a-d), BMF from grade 0 to grade 3(+++). (f), IHC, CD20. (g), IHC, Pax-5.

## Discussion

AMF, a variant of the myeloproliferative syndrome, is characterized by rapid onset of pancytopenia, normal red blood cell (RBC) morphology, extensive myelofibrosis, an absence of hepatosplenomegaly and a fatal outcome usually within months from diagnosis [5–8]. The patients had a typical clinical course of AMF, with a reticulin deposition in the bone marrow, no splenomegaly or fibrosis-related morphological changes in the RBC, rapid progression and a fatal outcome.

The patient has no JAK2 (Janus kinase 2, V617 F), MPL (thrombopoietin receptor, W515 K/L) and CALR (calreticulin, exon 9 indel) mutation which are identified in approximately 90% of patients to help with the diagnosis and the prognostic stratification of Primary myelofibrosis (PMF) patients [9,10]. This means that the cases had no basis of genetic mutations for PMF.

It is now generally accepted that the BMF observed inpatients with PMF is a ‘reactive’ inflammatory phenomenon affected by non-neoplastic cells in the bone marrow microenvironment [11]. Abnormal cytokine expression in myelofibrosis is believed to represent an inﬂammatory response [12]. Elevation of cytokines such as interleukin (IL)-1RA, IL-2,IL-6, IL-8, IL-12, IL-15, tumor necrosis factor α (TNF-α), macrophage inflammatory protein (MIP) 1α, interferon-γ (IFN-γ), and profibrogenic growth factors such as transforming growth factorβ (TGF-β), basic fibroblast growth factor (bFGF), vascular endothelial growth factor (VEGF), and Platelet-derived growth factor (PDGF) are considered to mediate BMF in patients with PMF^10−12^.

The cytokine profiles involved in CRS following CAR-T cells therapy encompass the effector cytokines such as IL-1RA, IL-1, IL-2, IL-4, IL-6, IL-8, IL-10, IL-12, IL-15, IL-18, TGF-β, IFN-α, IFN-γ, TNF-α, VEGF, MIP-1α, MIP-1β, soluble IL-2Rα, soluble IL-6R, granulocyte-macrophage colony-stimulating factor (GM-CSF), monocyte chemotactic protein (MCP)-1, ferritin, C-reactive protein (CRP) and MIP-1α, etc [4,13,14].

The cytokine profile is almost similar between BMF and CRS. It suggests that CRS might contribute to BMF. In our study, the patients who developed AMF had grade IV CRS with increased serum cytokine levels (i.e. IL-6) after CAR-T cells infusion. The patients’ bone marrow had tumor cells that means when CD19 CAR-T cells kill tumor cells in bone marrow [15] would induce high cytokine in bone marrow microenvironment, this would mediate AMF. In this case, before and after CD19 CAR-T cells infusion bone marrow biopsy showed the BMF has indicated a rapidly deteriorating from normal to grade III within a short period. These factors suggested that the cytokine profile of CRS might contribute to BMF.

## Conclusions

Despite the relapsed/refractory B-cell, ALL patient’s disease achieved CR post CD19 CAR-T cells therapy, he developed AMF in about 11 months after grade IV CRS. This suggests that the cytokine profile of CRS might contribute to AMF, but the mechanism remains elusive, further investigation is needed.
